# Tracking Carbapenem-Producing *Klebsiella pneumoniae* Outbreak in an Intensive Care Unit by Whole Genome Sequencing

**DOI:** 10.3389/fcimb.2019.00281

**Published:** 2019-08-08

**Authors:** Chen Chen, Yi Zhang, Sheng-Lei Yu, Yang Zhou, Si-Yu Yang, Jia-Lin Jin, Shu Chen, Peng Cui, Jing Wu, Ning Jiang, Wen-Hong Zhang

**Affiliations:** ^1^Department of Infectious Disease, Huashan Hospital of Fudan University, Shanghai, China; ^2^State Key Laboratory of Genetic Engineering and Institute of Biostatistics, School of Life Sciences, Fudan University, Shanghai, China; ^3^National Clinical Research Center for Aging and Medicine, Huashan Hospital of Fudan University, Shanghai, China

**Keywords:** carbapenemase-producing, *Klebsiella pneumoniae*, whole genome sequencing, outbreak, transmission

## Abstract

The presence of carbapenem-producing *Klebsiella pneumoniae* (CP-Kp) is a serious threat to the control of nosocomial infections. Plasmid-mediated horizontal transfer of the resistance gene makes it difficult to control hospital-acquired CP- Kp infections. Nine CP- Kp strains were isolated during an outbreak in the intensive care unit of Shanghai Huashan hospital in east China. We conducted a retrospective study to identify the origin and route of transmission of this CP-Kp outbreak. Whole-genome sequencing (WGS) analysis was performed on 9 clinical isolates obtained from 8 patients, and the results were compared to clinical and epidemiological records. All isolates were ST11 CP-Kp. Single-nucleotide polymorphisms and the presence and structure of plasmids indicated that this CP-Kp outbreak had different origins. These 9 isolates were partitioned into two clades according to genetic distance. Four plasmids, CP002474.1, CP006799.1, CP018455.1, and CP025459.1, were detected among the 9 isolates. The plasmid phylogeny and antibiotic resistance (AR) gene profile results were consistent with the sequencing results. We found that two clades of CP-Kp were responsible for this nosocomial outbreak and demonstrated the transmission route from two index patients. Plasmid carriage and phylogeny are a useful tool for identifying clades involved in disease transmission.

## Introduction

Carbapenem-producing *Klebsiella pneumoniae* (CP-Kp) refers to *K. pneumoniae* which is resistant to carbapenem with acquired carbapenemases. Despite commensal colonization in the respiratory tract, gastrointestinal tract, and skin, among other locations, *K. pneumoniae* is a typical nosocomial pathogen that causes high mortality. CP-Kp is a major concern for nosocomial infections worldwide. Infections caused by this pathogen are difficult to treat because CP-Kp is resistant to a wide variety of antibiotics, including carbapenems and colistin (Munoz-Price et al., 2013; Liu et al., 2016), which are last-resort drugs used in clinical practice. *K. pneumoniae* carbapenemases (KPCs) were first identified in the United States in 1996 (Munoz-Price et al., 2013). Furthermore, plasmid-mediated horizontal transfer of the resistance gene makes controlling hospital-acquired CP-Kp infection difficult (Conlan et al., 2014; Liu et al., 2016). Whole-genome sequencing (WGS) is a powerful tool for bacterial typing. In addition to use in pathogen identification, WGS is widely used to track epidemics and determine the origin and transmission route of nosocomial infections (Snitkin et al., 2012; Jiang et al., 2015; Onori et al., 2015; Cerqueira et al., 2017).

In recent years, rapid advancements in WGS have improved pathogen identification, including the identification of transmission routes and evolutionary patterns, as a genotyping method that is easily accessible. Drug-resistance mechanisms and the potential transfer of antibiotic resistant genes by plasmids has also been analyzed by WGS. However, conventional short-read sequencing produces inaccurate genome assemblies which cannot be used to identify pathogens and track outbreak origins. Moreover, it is difficult to confirm the presence of plasmids and mobile genetic elements through short-read sequencing. In long-read sequencing, plasmid-mediated resistance, and insertion sequence variation can be analyzed. Phylogenomic analysis, together with molecular characterization of drug resistance determinants and plasmids, enables determination of the polyphyletic origins of CP-Kp strains and detection of common genetic traits in carbapenem-resistant strains. In this study, we evaluated the transmission route of CP-Kp in a tertiary hospital and attempted to identify the origin of this outbreak.

## Methods

### Patients and Isolates

We analyzed 8 patients with CP-Kp infection who were admitted to the Department of Infectious Diseases at Huashan Hospital from December 2016 to April 2017. The CP-Kp outbreak occurred in a new infectious disease care unit (IDCU), a 5-ward (17 beds) intensive care center, which was operational only for 2 months. A total of 9 *K. pneumoniae* strains were isolated from the blood, sputum, urine, and exudate from a deep venipuncture site. Clinical isolates from the same sites showing the same antibiotic susceptibility test (AST) result were not included in this study. ASTs, carbapenemase gene identification, and WGS analysis were performed for each isolate. The clinical records and laboratory test results of patients were extracted from the patient administration system. The observation endpoint was defined as discharge from the hospital or death. Additionally, we carried out environment sampling and collected hand swabs from the medical staff. A waiver of informed consent was granted by the Huashan Hospital ethical committee for this study.

### Antimicrobial Susceptibility Testing and Carbapenemase Gene Identification

ASTs were carried out for all CP-Kp outbreak isolates using the disk-diffusion method, and the susceptibility breakpoint was interpreted as recommended by the Clinical and Laboratory Standards Institute, version 2017. The tested antibiotics included amikacin, gentamicin, piperacillin/tazobactam, ceftolozane/tazobactam, cefazolin, cefuroxime, ceftazidime, cefepime, imipenem, ciprofloxacin, trimethoprim/sulfamethoxazole, and meropenem. A strain was identified as carbapenem-resistant when the inhibition zone of imipenem or meropenem was smaller than 19 mm in diameter. We also tested for the presence of carbapenemase gene (*bla*_KPC_, *bla*_NDM−1_, and *bla*_OXA−48_) by multiplex PCR (Poirel et al., 2011) (Supplementary Material 1).

### Whole-Genome Sequencing and Plasmid Confirmation

Total DNA was extracted from the 9 CP-Kp isolates using the TIANAmp Micro DNA Kit (TIANAmp, Tiangen Biotech, Tiangen, China) according to the manufacturer's recommendations. After synthesizing second-strand DNA, DNA libraries were constructed by DNA-fragmentation, end-repair, A-tail addition, adapter-ligation, and PCR amplification. An Agilent 2100 Bioanalyzer (Agilent, Santa Clara, CA, USA) was used for quality control of the DNA libraries.

WGS was performed on a Nova-seq platform (Illumina, San Diego, CA, USA) by constructing a paired-end (PE) library with average insertion lengths of 300–500 base pairs (bp). Raw sequencing data (2 Gb/sample) was pre-processed as follows: (1) removing adapter sequences, (2) removing reads with over 20 bp of low quality (Phred quality score <20), (3) removing reads with over 20 bp of ambiguous bases, and (4) removing duplicated reads. *De novo* assembly was performed using SPADES v3.11.1. Reads were also aligned to the reference isolate *K. pneumonia*e SWU01 (Genbank accession number CP018454.1; downloaded and annotated through NCBI) using bowtie2 (Langmead and Salzberg, 2012). The average depth of Illumina was ~300, and the mapped ratio was 87.60% on average with 32,089,923 total reads. The multilocus sequences were identified using the pMLST 1.4 database (https://cge.cbs.dtu.dk/services/MLST/). Antimicrobial resistance genes were identified and located using Resfinder tool 2.1 and the BIGSDB database (http://bigsdb.pasteur.fr/). Detailed information regarding Illumina short-read sequencing of the CP-Kp outbreak isolates including total reads, mapped reads, and mapped ratio is shown in Supplementary Material 2.

A sequencing library of the CP-Kp outbreak strains, KP-s1, KP-s5, and KP-s7, were prepared as described previously for GridION nanopore (Oxford, UK) sequencing. The average depth for Nanopore is ~400 and the error rate is ~15%. Unicycling was carried out to combine the short Illumina reads and long Nanopore reads (Phillippy et al., 2017). We annotated the chromosomes and plasmids using the BASys annotation system (https://www.basys.ca). All sequence data in this study have been deposited in GeneBank under Bioproject number PRJNA551327.

### Single-Nucleotide Polymorphism (SNP) Analysis and Phylogenetic Tree Construction

SNP analysis of the isolates was conducted using SAMtools (Li et al., 2009). The phylogenetic tree was constructed according to the diversity of the core genome SNPs. The evolutionary distances were calculated based on the similarity of the SNP genotyping and validated by bootstrap analysis. All positions containing gaps and missing data were eliminated. We employed Bayesian evolutionary analysis in BEAST v 1.10.4 (Drummond et al., 2002) to estimate the coalescence time based on strict molecular clock models. The result was used to construct the phylogenetic tree in FigTree v 1.4.4 (Morariu et al., 2008).

## Results

### Overview of *K. pneumoniae* Outbreak

The index case (Pt-1) of this outbreak was identified to have CP-Kp infection on December 2016. Eight days prior, Pt-1 was admitted into the IDCU. He was sent to the emergency room and underwent endotracheal intubation, assisted ventilation, and urethral catheterization. After a 24-h stay in the emergency room, he was transferred to an isolation ward of the IDCU after being diagnosed with H7N9 influenza A infection by traditional PCR. No CP-Kp was detected in the blood upon admission to the isolation ward but blood-stream CP-Kp was reported on December 27 after he was transferred to ward 1 in the intensive care unit.

**Figure 1:**
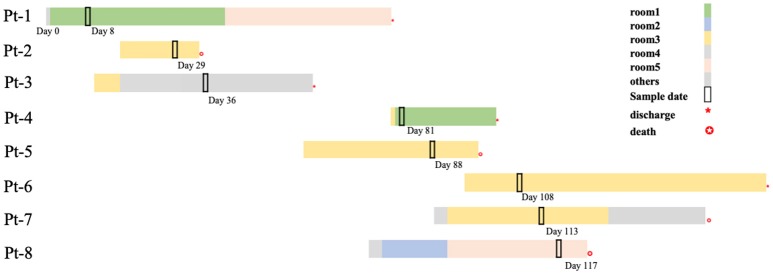
Timelines and overlap of outbreak CP-Kp isolates. Day 0 refers to the day of admission to our hospital of Pt-1. Sample days are noted nearby the black box indicating the culture-positive date.

In the next 4 months, another 7 patients were continuously confirmed to have CP-Kp infection in the IDCU (Table 1). The overlap period was defined as the time shared by the patients in the same ward room (Snitkin et al., 2012). As shown in Figure 1, an overlap period between Pt-4 and Pt-5 was observed in Ward 3, and Pt-5, Pt-6, and Pt-7 shared an overlap period during this outbreak. Notably, Pt- 2, Pt-3, Pt-4, Pt-5, Pt-6, and Pt-7 were all admitted to Ward 3 in the IDCU, while Pt-1 and Pt-8 were not (Figure 1).

**Table 1:** Baseline characteristics and outcomes of patients enrolled in CP-Kp outbreak.

**Patient**	**Isolate**	**Gender**	**Age**	**Underlying diseases**	**Sample date since admission(d)**	**Culture Site**	Treatment therapy^*^	**Glucocorticoids use before culturing positive**	**Outcome**	**Cause of death**
Pt-1	KP-s1	M	45	H7N9 influenza A infection	8	Blood	MEM, TIG, FOS, MIF	Yes	Improved	/
Pt-2	KP-s2	M	65	Severe pneumonia	12	Sputum	MEM, TIG, AMI, LZD, VRC, CRO	No	Died	CP-kp infection
Pt-3	KP-s3	M	51	Hepatitis B; decompensated liver cirrhosis	25	Blood	TZP, AMI, LEV	Yes	Improved	/
Pt-4	KP-s4	F	52	Severe pneumonia	2	Sputum	I/C, VAN, MXF, CAS	No	Improved	/
Pt-5	KP-s5	F	35	Central nervous system infection	29	Urine	MEM, LZD, INH, RFP, PZA, EMB	Yes	Died	Central nervous system infection
	KP-s6					Sputum				
Pt-6	KP-s7	M	30	Acute on chronic liver failure	12	Blood	MEM, AMI, FOS, TIG, CAS, MIF	Yes	Improved	/
Pt-7	KP-s8	M	54	Acute on chronic liver failure	24	Urine	MEM, LZD, MXF, MIF	Yes	Died	CP-kp infection
Pt-8	KP-s9	M	73	Acute severe hepatitis E	43	Exudate	MEM,MXF,TIG,FOS,CAS,MIF	Yes	Died	CP-kp infection

* *AMI, Amikacin; CRO, Ceftriaxone; TZP, Piperacillin/tazobactam; LEV, Levofloxacin; MXF, Moxifloxacin; I/C, Imipenem/cilastatin; MEM, Meropenem; FOS, Fosfomycin; TIG, Tigecycline; VAN, Vancomycin; LZD, linezolid; VRC, Voriconazole; MIF, Micafungin; CAS, Caspofungin; INH, Isoniazid; RFP, Rifampicin; PZA, Pyrazinamide; EMB, Ethambutol*.

No epidemiologic and pathogenic evidence indicated environmental involvement or the medical staff in the transmission of nosocomial CP-Kp. Apart from active screening of the medical environment and staff, nosocomial infection control measures were implemented to end this outbreak; the measures included isolating the patients with infectious CP-Kp, contact precautions, and enforcing hand hygiene and environmental cleaning. Finally, among the 8 patients involved in this outbreak, 4 patients gradually recovered (50.00%), three patients died of CP-Kp infection (37.50%), and one patient died of central nervous system infection (12.50%).

### Antibiotic Susceptibility and Characterization of AR Genes

**Figure 2:**
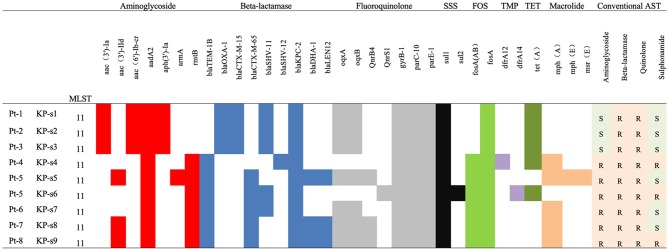
Antibiotic susceptibility and characterization of AR genes among CP-Kp isolates. SSS, Sulphonamides; FOS, Fosmycin; TMP, Trimethoprim; TET, Tetracycline.

All *K. pneumoniae* isolates from the 8 patients were multi-drug resistant. As indicated in Figure 2 and Supplementary Material 3, isolates KP-s1, KP-s2, and KP-s3 were susceptible to aminoglycoside and sulfonamide. Isolates KP-s5, KP-s7, and KP-s8 were sensitive to sulfonamide while the other 3 isolates were resistant to all antibiotics tested. According to the susceptibility results, 9 isolates were all resistant to meropenem, imipenem, cefepime, ceftazidime, cefotaxime, cefuroxime, cefazolin, ceftolozane/tazobactam, and piperacillin/tazobactam (100.00%; Supplementary Material 4). Resistance to amikacin and gentamicin was detected in 6 of the 9 isolates (66.67%), and trimethoprim/sulfamethoxazole resistance was confirmed in 3 isolates (33.33%). Based on the WGS results, we further characterized the AR genes among the 9 isolates. All isolates were confirmed to possess genes for resistance to aminoglycoside, beta-lactamase, fluoroquinolone, sulfonamide, and formycin. KP-s4, KP-s6 carried a trimethoprim resistance gene, while a tetracycline resistant gene was found in KP-s1, KP-s2, KP-s3, KP-s4, and KP-s6. Macrolide-resistant genes were detected in 5 isolates.

### Comparison of Isolates According to WGS-Based SNPs

**Figure 3:**
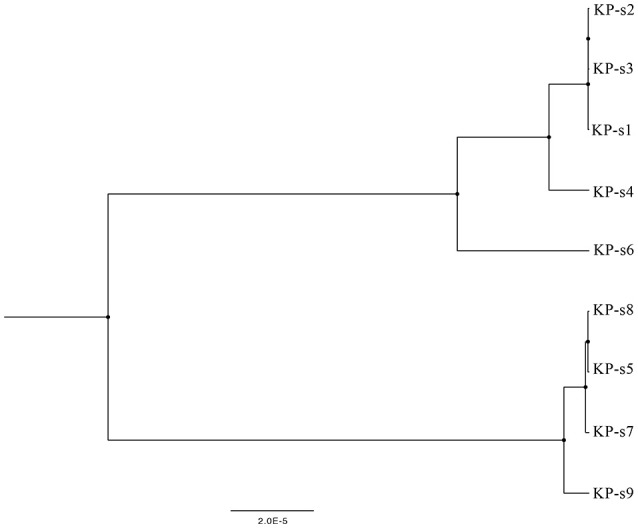
Phylogenetic tree of CP-Kp outbreak isolates based on whole-genome sequencing. The upper branch includes KP-s1, s2, s3, s4, and s6, KP-s5, s7, s8, and s9 are in the lower branch.

**Figure 4:**
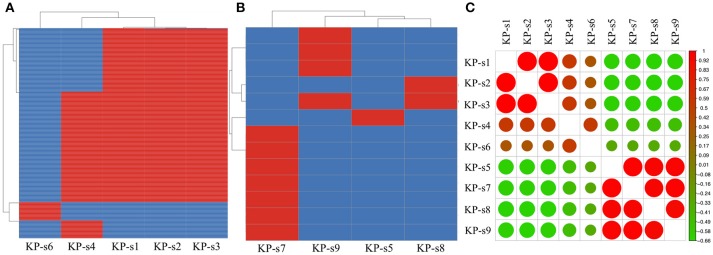
**(A)** SNP analysis in isolate KP-s1, KP-s2, KP-s3, KP-4, and KP-s5. One SNP was found within Clade 1. **(B)** SNP analysis in Clade 2 (isolate KP-s5, KP-s7, KP-s8, and KP-s9). A total of 13 SNPs existed within Clade 2. **(C)** Correlation heatmap of CP-Kp outbreak isolates.

A total of 1,545 SNPs were detected in the 9 isolates (Supplementary Material 5). The evolutionary relationships of the 9 CP-Kp isolates are presented in Figure 3. The isolates involved in this nosocomial CP-Kp outbreak were partitioned into two distinct clades. The first clade (Clade 1), encompassing isolates KP-s1, KP-s2, and KP-s3, contained only one SNP, with extremely high homology observed in the three strains (Figures 3, 4A). Isolates KP-s5, KP-s7, KP-s8, and KP-s9 were clustered into Clade 2, in which 13 SNPs were identified (Figures 3, 4B). Among them, KP-s5 and KP-s6, collected from Pt-5, belonged to different SNP clades. Higher genomic similarity between Clade 1 and isolate KP-s4 than isolate KP-s6 was observed in the phylogenetic tree. Additionally, all isolates were categorized as ST11 type by comparisons with the pMLST database. The correlation among the 9 isolates is shown in Figure 4C, and the high rate of similarity within Clades 1 and 2 was further confirmed. Few regions were non-covered in our 9 sequenced samples, which were considered as “gaps.” Comparative analyses of the genome sequence revealed that more than 95% of the reference sequences could be deeply mapped (Supplementary Material 6). The detailed information and function of the inconsistent “gaps” are listed in Supplementary Materials 6, 7.

To identify the differences between the CP-Kp outbreak isolates, we performed SNP annotation analysis (Supplementary Material 7). The only SNP (chromosome CP018454.1, 1508195 G>T) observed in Clade 1 was in the gene related to the putative uncharacterized protein/putative membrane-anchored protein (ydbH). In total, 13 SNPs were observed in isolates KP-s5, KP-s7, KP-s8, and KP-s9, and some of their functions remain unknown (Supplementary Material 7).

### Comparative Plasmid Analysis

**Figure 5:**
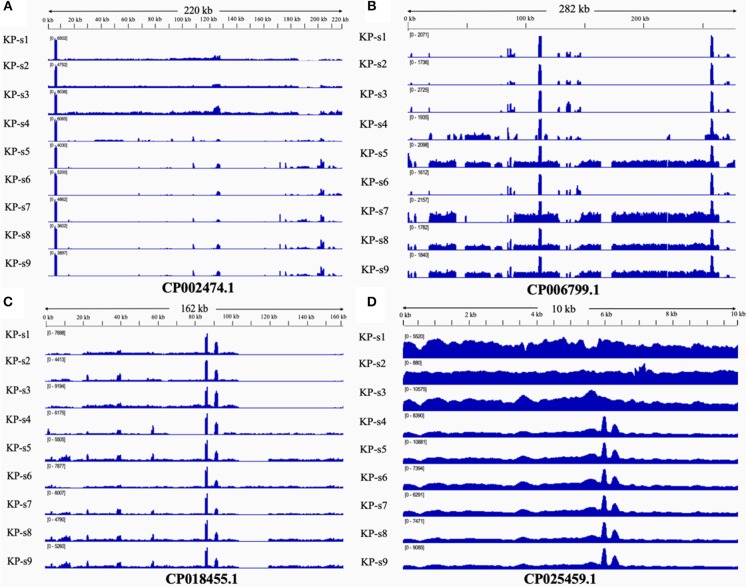
**(A)** Mapping of coverage of plasmid CP002474.1 in CP-Kp outbreak isolates. Plasmid CP002474.1 was found in Clade 1. **(B)** Mapping of coverage of plasmid CP006799.1 in CP-Kp outbreak isolates. Plasmid CP006799.1 was mapped to part of KP-s4, s6, and Clade 2 (isolate KP-s5, KP-s7, KP-s8, and KP-s9). **(C)** Mapping of coverage of plasmid CP018455.1 (*Klebsiella pneumonia*e strain SWU01 plasmid unnamed complete sequence) in CP-Kp outbreak isolates. All 9 isolates mapped to plasmid CP018455.1. **(D)** Mapping of coverage of plasmid CP025459.1 in CP-Kp outbreak isolates. Plasmid CP025459.1 was detected in all CP-Kp outbreak isolates.

The presence and characteristics of the plasmids detected in the 9 CP-Kp isolates were consistent with the WGS analysis results. Plasmids from isolates in Clade 1 were mapped to CP002474.1, CP018455.1, and CP025459.1. Plasmids CP006799.1, CP018455.1, and CP025459.1 were mapped to Clade 2. Plasmids from isolates KP-s4 and KP-s6 were partially mapped to plasmid CP018455.1; these two isolates were found to contain shortened plasmid reads of CP006799.1. Among the 4 mapped plasmids, 3 carried resistance genes (Table 2). Plasmid CP002474.1 was found to exist in Clade 1, as shown in Figure 5A. Mapped to KP-s4, s6, and Clade 2, plasmid CP006799.1 was shown to carry the NDM-1 gene, while the coordinate sequence part (7770-8582) was missing from all 9 isolates (Figure 5B). Notably, the isolate KP-s5 showed a longer plasmid CP006799.1 sequence than the other cluster 2 isolates, ranging from 270 to 290 kb. CP018455.1, a *K. pneumonia*e strain SWU01 plasmid unnamed complete sequence, is available in GenBank but is not annotated. This is the blaKPC2 plasmid (coordinates: 21518–22399). A missing fragment from 103 to 162 kb of plasmid CP018455.1 in Clade 1 was also observed (Figure 5C). Moreover, a fragment was missing from ~65 to 85 kb of isolate KP-s4 and from 103 to 120 kb of isolate KP-s5. Plasmid CP025459.1 was detected in all CP-Kp outbreak isolates, showing 100% coverage in this CP-Kp outbreak (Figure 5D).

**Table 2:** Characteristics of mapped plasmids through comparative plasmid analysis.

**NCBI GenBank No**.	**Plasmid Name**	**Size (kb)**	**G+C content (%)**	**Resistance Gene**
CP002474.1	pUUH239.2	220.824	52.8185	aadA2 mph(A) sul1 dfrA12 tet(A) catB3 blaTEM-1B blaCTX-M-15 blaOXA-1 aac(6′)Ib-cr
CP006799.1	Plasmid1	283.371	46.4105	aadA2 armA aph(3′)-VIa sul1 mph(E) QnrB1 dfrA14 dfrA12 msr(E) catB3 blaOXA-1 blaNDM-1 aac(6′)Ib-cr
CP018455.1	Unnamed	162.552	53.6345	rmtB blaSHV-12 blaTEM-1B blaKPC-2 blaCTX-M-65
CP025459.1	p69-3	10.06	55.0596	None

*A total of four plasmids including CP002474.1, CP006799.1, CP018455.1, and CP025459.1 (GenBank number) were mapped to in this study*.

## Discussion

In this study, we retrospectively investigated a nosocomial CP-Kp outbreak and determined the transmission pattern during this hospital-acquired outbreak by WGS analysis using Illumina short-read sequencing and Nanopore long-read sequencing.

Although multilocus sequence typing (MLST) and pulsed-field gel electrophoresis are well-known genotyping methods (Zhan et al., 2017), the shared MLST did not reveal differences between these 9 CP-Kp isolates. Furthermore, despite its wide use in outbreak investigation, pulsed-field gel electrophoresis is associated with non-reproducible results, bias, and long turn-around time (Hammerum et al., 2015).

Traditional molecular typing techniques are limited in detecting minor genomic differences between isolates. WGS analysis, however, can overcome this limitation and is widely used for pathogen identification and tracking the origin of outbreaks, particularly in cases of nosocomial infections. By using Illumina short-read sequencing and Nanopore long-read sequencing, CP-Kp isolates from two separate clades were detected in this outbreak and the putative transmission route was reconstructed.

Nine isolates belonging to two transmission routes were identified in this CP-KP outbreak. The index patient of transmission 1 was Pt-1, and the infection was transmitted to Pt-2 and Pt-3 (isolates KP-s2 and KP-s3) from the same phylogenetic sub-clade. Moreover, Pt-2 and Pt-3 had overlapping IDCU stays with Pt-1, and the 3 isolates from these patients exhibited considerable genomic similarity. *Klebsiella pneumoniae* was detected in the blood culture of Pt-3 1 week after he was transferred to another ward, while KP-s3 still belonged to Clade 1. Although there was a gap of more than 1 month between the confirmation of CP-Kp infection in Pt-3 and Pt-4, the CP-Kp isolates from both patients shared relatively high similarity, indicating the persistence and minor evolution of KP-s4.

The existence of Clade 2 indicates a separate transmission route of CP-Kp infection in Pt-5, Pt-6, Pt-7, and Pt-8. This infection may have originated from Pt-5, who was transferred from another hospital, and there was a 28-day interval between the CP-Kp transmission from to Pt-5 to Pt-6, Pt-7, and Pt-8. SNP analysis of Clade 2 indicated that Pt-5 transmitted the infection to both Pt-6 and Pt-7, and CP-Kp was then transmitted from Pt-7 to Pt-8 (isolate KP-s9). Isolate KP-s6 from Pt-5 was not included in this transmission.

Among the 8 patients involved in this outbreak, 6 had been administered glucocorticoid treatment and all patients were administered antimicrobial therapy prior to receiving culture-positive results. Studies have reported that admission to the ICU, carbapenem treatment, and glucocorticoids as risk factors for acquiring CP-Kp infection (Giannella et al., 2014; Cano et al., 2018). In immunocompromised patients with severe underlying diseases, additional prevention and control measures are needed.

All 9 clinical isolates carried *bla*_*KPC*−2_ and were multi-drug resistant. Analysis of the antimicrobial gene revealed that the presence of the aminoglycoside- and sulfonamide-resistant genes were inconsistent with the results of phylogeny and phenotype analysis (conventional AST). Similar results have been obtained in other studies (Crofts et al., 2017; Tamma et al., 2019). Minimum inhibitory concentration analysis or conventional AST can help decide which antibiotics should to be used, while genes confirmed by WGS lack a standard antibiotic gene database (Balloux et al., 2018; Greninger, 2018; Tamma et al., 2019). Future studies are required to further investigate the underlying causes of the inconsistency in the resistance profile, phylogeny, and phenotype.

Short-read sequencing alone cannot reveal whether the genome scaffolds originated from the chromosome or plasmid. Several studies have demonstrated the advantage of Nanopore long-read sequencing in identifying horizontal transfer of plasmids and tracking plasmid diversity (Conlan et al., 2014, 2016; Gorrie et al., 2018). A recent study showed that suspected carbapenem-resistant *Enterobacter cloacae* transmission occurred through non-overlapping plasmids (Chen et al., 2017). Genomic evidence, including a shared drug-resistance gene, cannot always reveal the route of transmission because of the existence of plasmid-mediated CP-Kp transmission. In our study, the presence of plasmid characteristics was consistent with the results of core genome analysis, indicating that the presence of plasmids can help determine transmission clusters and routes. Plasmid analysis may be a useful supplementary method during outbreak investigation and can be applied in nosocomial infection control.

Notably, isolates KP-s5 and KP-s6 from Pt-5 were genetically confirmed as different strains. This indicates that this patient was infected with two CP-Kp strains simultaneously. These results further confirmed co-infection with different genetic subtypes of the pathogen, and emphasize the need for targeted antibiotic application and treatment regimens, given the existence of diverse carbapenem resistance mechanisms (Zhu et al., 2015).

During this nosocomial CP-Kp outbreak, our IDCU and Department of Hospital-Acquired Infection established infection control measures to terminate the outbreak in the intensive care unit. Several measures, as recommended by the World Health Organization, were implemented to eliminate the nosocomial infection. Apart from isolating the *K. pneumoniae*-infected patients, we assigned a specialized nursing staff, enforced hand hygiene, used caution in contact, and performed environmental cleaning. The *K. pneumoniae* nosocomial infection was successfully limited to 8 patients. In June 2017, this CP-Kp outbreak ended.

There were several limitations to this study. First, this retrospective study could not provide immediate assistance to effectively control CP-Kp. Real-time tracking and whole-genome sequencing are urgently required to improve nosocomial infection surveillance and management methods. Second, we failed to obtain a positive *K. pneumoniae* sample from the medical staff or environment. Whether medical personnel and the environment were involved in this outbreak remains uncertain. Third, because of the limited sample size and study duration, the putative transmission route may not represent the CP-Kp infection trend in the entire hospital, and further multi-center studies should be carried out.

## Conclusion

Two clades of CP-Kp were identified during this nosocomial outbreak. Combined application of Illumina short read and Nanopore long-read sequencing can be used to track nosocomial infections and identify transmission routes. Real-time surveillance of nosocomial outbreaks can help identify the origin of infection and detailed transmission route, which will facilitate the timely control of such outbreaks.

## Data Availability

All datasets generated for this study are included in the manuscript and the Supplementary Files.

## Author Contributions

CC and YZha were sub-investigator in this research, collected and analyzed the clinical, and sequencing data and wrote the paper. S-LY and YZho contributed in the help of analysis of clinical information and wrote the paper. S-YY and PC were sub-investigators in performing the antibiotic resistance gene confirmation. J-LJ and SC contributed in clinical assistance and infection control for this outbreak. NJ and JW were investigators who performed bioinformatic analyses. W-HZ is the head of the Department of Infection Diseases and principal investigator in the whole genome sequencing research, and supervised data collection and analysis and paper draft.

### Conflict of Interest Statement

The authors declare that the research was conducted in the absence of any commercial or financial relationships that could be construed as a potential conflict of interest.
