# Supplementary material for: Tracking Carbapenem-Producing Klebsiella pneumoniae Outbreak in an Intensive Care Unit by Whole Genome Sequencing
Source: Front Cell Infect Microbiol. 2019 Aug 8;9:281. doi: 10.3389/fcimb.2019.00281 (PMC6694789; doi:10.3389/fcimb.2019.00281)
Supplement: Supplementary file 1 [file Data_Sheet_1.docx]

**Revised Frontiers-472219**

**Supplementary information**

**Tracking carbapenem-producing *Klebsiella pneumoniae* outbreak in an intensive care unit by whole genome sequencing**

**Authors (email address)**

Chen Chen^1*^(atheran@163.com), Yi Zhang^1*^(Atobezy@163.com), Sheng-Lei Yu ^1*^（[susan0156241@126.com](mailto:susan0156241@126.com)）, Yang Zhou^1^（[zhouyang_yz@fudan.edu.cn](mailto:zhouyang_yz@fudan.edu.cn)）, Si-Yu Yang^1^([rainis777@163.com](mailto:rainis777@163.com)), Jia-Lin Jin^1^([jinjialin@fudan.edu.cn](mailto:jinjialin@fudan.edu.cn)), Shu Chen^1^(hschenshu@163.com), Peng Cui^1^(keanuc@163.com), Jing Wu^1#^(jingee@fudan.edu.cn), Ning Jiang^3#^(Ningjiang@fudan.edu.cn), Wen-Hong Zhang^1,2#^(zhangwenhong@fudan.edu.cn; wenhongzhang_hs@126.com)

Chen Chen, Yi Zhang and Sheng-Lei Yu contributed equally to this essay.

**Affiliations:**

1. Department of infectious disease, Huashan Hospital of Fudan University, Shanghai 200040, China
2. National Clinical Research Center for Aging and Medicine, Huashan Hospital, Fudan University, Shanghai 200040, China
3. State Key Laboratory of Genetic Engineering and Institute of Biostatistics, School of Life Sciences, Fudan University, Shanghai 200433, China

**Running title:** Tracking carbapenem-resistant *Klebsiella pneumoniae* outbreak by WGS

**#Correspondence to:**

Wen-Hong Zhang, Department of Infectious Diseases, Huashan Hospital of Fudan University, Shanghai 200040. Mailing address: 12 Wulumuqi Zhong Road, Shanghai 200040, China. Tel: +86-21-52888123. Fax: +86-21-62489015. Email: [zhangwenhong@fudan.edu.cn](mailto:zhangwenhong@fudan.edu.cn); [wenhongzhang_hs@126.com](mailto:wenhongzhang_hs@126.com).

Ning Jiang, Department of State Key Laboratory of Genetic Engineering and Institute of Biostatistics, School of Life Sciences, Fudan University, Shanghai 200433, China Email: Ningjiang@fudan.edu.cn

Jing Wu, Department of Infectious Diseases, Huashan Hospital of Fudan University, Shanghai 200040. Mailing address: 12 Wulumuqi Zhong Road, Shanghai 200040, China. Email: [jingee@fudan.edu.cn](mailto:jingee@fudan.edu.cn)

**Supplementary material**

Supplementary material 1. Multiplex PCR primers (*bla*_KPC_, *bla*_NDM-1_, and *bla*_OXA-48_) and electrophoregram.

Supplementary material 2. The detailed information of Illumina short-read sequencing of CP-Kp outbreak isolates including total reads, mapped reads and mapped ratio.

Supplementary material 3. Conventional antimicrobial susceptibility test results of CP-Kp outbreak isolates by disk-diffusion method.

Supplementary material 4. The antimicrobial susceptibility proportion of CP-Kp outbreak isolates.

Supplementary material 5. SNPs among CP-Kp outbreak isolates. A total of 1545 SNPs were detected from 9 isolates.

Supplementary material 6. Few regions were non-covered in our 9 sequenced samples, which were considered as ‘gaps’. Comparative analyses of the genome sequence revealed that more than 95% of the reference sequences could be deeply mapped. Only few regions were non-covered in our 9 sequenced samples, which were considered as ‘gaps’. Though relatively high coverage, there were still some ‘gaps’.

Supplementary material 7. Functional annotation of 13 SNPs among clade 2 (isolate KP-s5, KP-s7, KP-s8 and KP-s9).

Supplementary material 1 Multiplex PCR primers (*bla*_KPC_, *bla*_NDM-1_, and *bla*_OXA-48_) and electrophoregram.

| Primers | （5‘－3’） | Size（bp） |
| --- | --- | --- |
| KPC-F | CGTCTAGTTCTGCTGTCTTG | 798 |
| KPC-R | CTTGTCATCCTTGTTAGGCG |  |
| NDM-F | GGTTTGGCGATCTGGTTTTC | 621 |
| NDM-R | CGGAATGGCTCATCACGATC |  |
| OXA-F | GCGTGGTTAAG GATGAACAC | 438 |
| OXA-R | CATCAAGTTCAACCCAACCG |  |


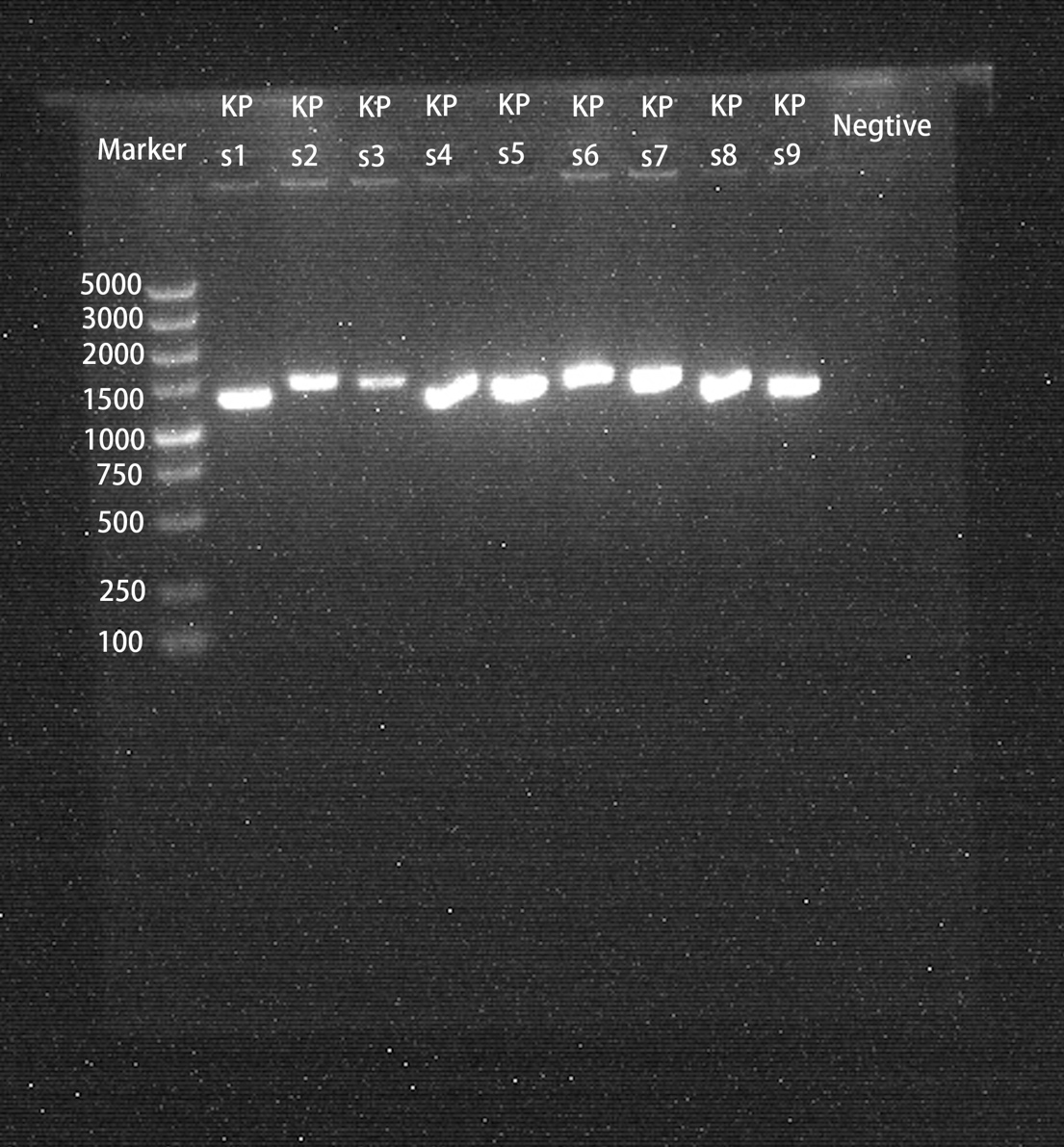


Supplementary material 2 The detailed information of Illumina short-read sequencing of CP-Kp outbreak isolates including total reads, mapped reads and mapped ratio.

| sample | Total reads | Total mapped | Mapped ratio (%) |
| --- | --- | --- | --- |
| KP-s1 | 39475318 | 35605207 | 90.20% |
| KP-s2 | 20532922 | 19389438 | 94.43% |
| KP-s3 | 48783514 | 42049427 | 86.20% |
| KP-s4 | 36530500 | 30786965 | 84.28% |
| KP-s5 | 26268078 | 22798832 | 86.79% |
| KP-s6 | 31405426 | 26852767 | 85.50% |
| KP-s7 | 30995762 | 26746292 | 86.29% |
| KP-s8 | 26689468 | 23301532 | 87.31% |
| KP-s9 | 28128324 | 24574226 | 87.36% |

Supplementary material 3 Conventional antimicrobial susceptibility test results of CP-Kp outbreak isolates by disk-diffusion method.

| Patient ID | Isolate ID | AMI | GEN | PRL | KZ | CXM | CTX | CAZ | FEP | CIP | SXT | SCF | TZP | I/C | MEM |
| --- | --- | --- | --- | --- | --- | --- | --- | --- | --- | --- | --- | --- | --- | --- | --- |
| Pt-1 | KP-s1 | 20 | 20 | 6 | 6 | 6 | 6 | 6 | 6 | 6 | 17 | 6 | 6 | 6 | 6 |
| Pt-2 | KP-s2 | 19 | 20 | 6 | / | 6 | 6 | 6 | 6 | 6 | 19 | 6 | 6 | 8 | 6 |
| Pt-3 | KP-s3 | 17 | 19 | 6 | 6 | 6 | 6 | 6 | 6 | 6 | 18 | 6 | 6 | 6 | 6 |
| Pt-4 | KP-s4 | 6 | 6 | 6 | 6 | 6 | 6 | 6 | 6 | 6 | 6 | 6 | 6 | 6 | 6 |
| Pt-5 | KP-s5 | 6 | 6 | 6 | 6 | 6 | 6 | 6 | 6 | 6 | 13 | 6 | 6 | 6 | 6 |
|  | KP-s6 | 6 | 6 | 6 | 6 | 6 | 6 | 6 | 6 | 6 | 6 | 6 | 6 | 6 | 6 |
| Pt-6 | KP-s7 | 6 | 6 | 6 | 6 | 6 | 6 | 6 | 6 | 6 | 12 | 6 | 6 | 6 | 6 |
| Pt-7 | KP-s8 | 6 | 6 | 6 | 6 | 6 | 6 | 6 | 6 | 6 | 14 | 6 | 6 | 6 | 6 |
| Pt-8 | KP-s9 | 6 | 6 | 6 | 6 | 6 | 6 | 6 | 6 | 6 | 6 | 6 | 6 | 6 | 6 |

Abbreviations: AMI, Amikacin; GEN, Gentamicin; PRL, Piperacillin; KZ, Cefazolin; CXM, Cefuroxime; CTX, Cefotaxime; CAZ, Ceftazidime; FEP, Cefepime; CIP, Ciprofloxacin; SXT, Trimethoprim/sulfamethoxazole; SCF, cefoperazone/sulbactam; TZP, Piperacillin/tazobactam; I/C, Imipenem / cilastatin; MEM, meropenem.

| **Drug Classification** | **Antibiotics Tested** | **Resistant** | **Intermediate** | **Susceptible** |
| --- | --- | --- | --- | --- |
| Aminoglycoside | Amikacin | 66.7% | 0.0% | 33.3% |
|  | Gentamicin | 66.7% | 0.0% | 33.3% |
| Beta-lactam/Beta-lactamase inhibitor | Piperacillin/tazobactam | 100.0% | 0.0% | 0.0% |
|  | Ceftolozane/tazobactam | 100.0% | 0.0% | 0.0% |
| Cepholosporin (1st & 2nd generation) | Cefazolin | 100.0% | 0.0% | 0.0% |
|  | Cefuroxime | 100.0% | 0.0% | 0.0% |
| Cepholosporin (3rd & 4th generation) | Cefotaxime | 100.0% | 0.0% | 0.0% |
|  | Ceftazidime | 100.0% | 0.0% | 0.0% |
|  | Cefepime | 100.0% | 0.0% | 0.0% |
| Carbapenem | Imipenem | 100.0% | 0.0% | 0.0% |
|  | Meropenem | 100.0% | 0.0% | 0.0% |
| Fluoroquinolone | Ciprofloxacin | 100.0% | 0.0% | 0.0% |
| Folate synthesis inhibitor | Trimethoprim/sulfamethoxazole | 33.3% | 0.0% | 66.7% |

Supplementary material 4 The antimicrobial susceptibility proportion of CP-Kp outbreak isolates

Supplementary material 5. SNPs among CP-Kp outbreak isolates. A total of 1545 SNPs were detected from 9 isolates.


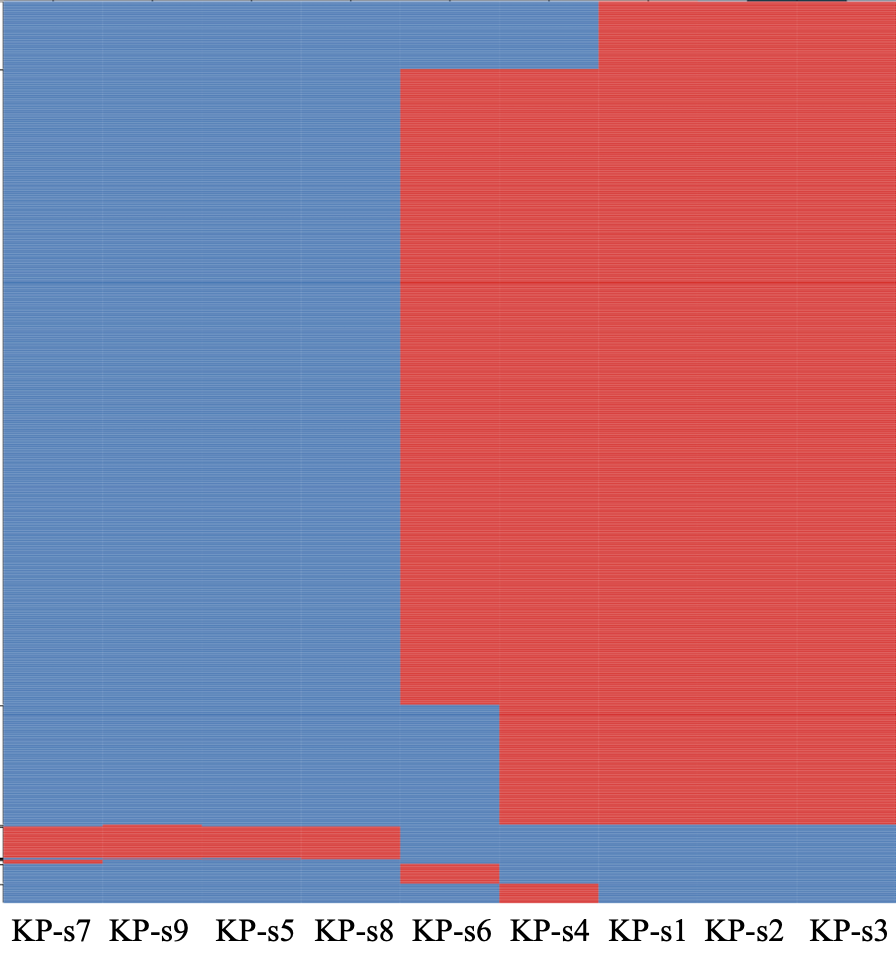


Supplementary material 6. Few regions were non-covered in our 9 sequenced samples, which were considered as ‘gaps’. Comparative analyses of the genome sequence revealed that more than 95% of the reference sequences could be deeply mapped. Only few regions were non-covered in our 9 sequenced samples, which were considered as ‘gaps’. Though relatively high coverage, there were still some ‘gaps’.


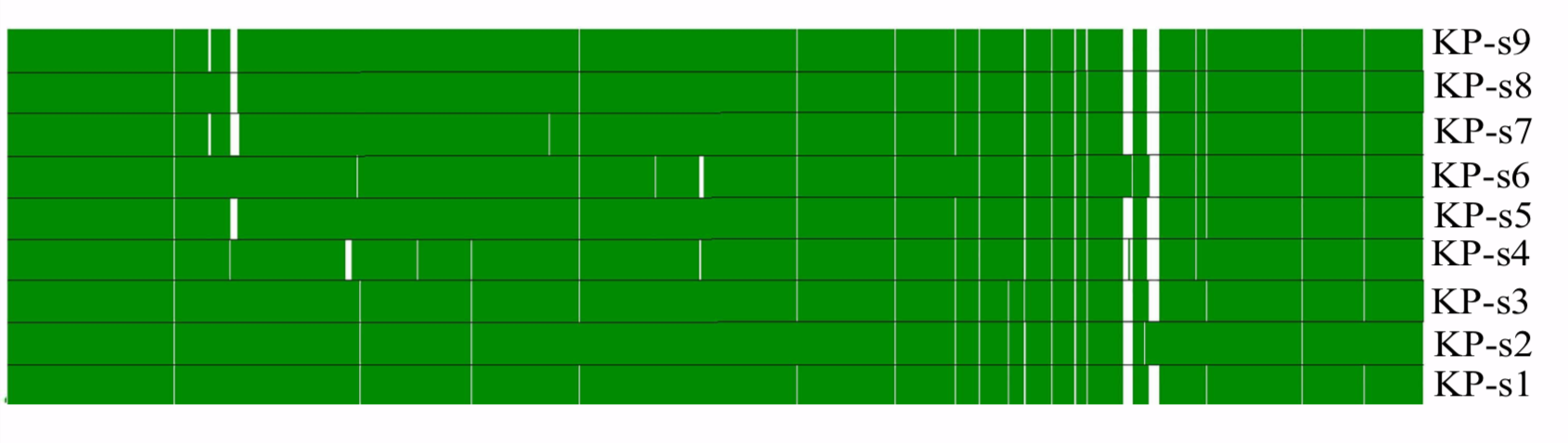


Supplementary material 7. Functional annotation of 13 SNPs among clade 2 (isolate KP-s5, KP-s7, KP-s8 and KP-s9).

| **Chr** | **Position** | **Ref** | **Alt** | **KP-s5** | **KP-s7** | **KP-s8** | **KP-s9** | **contig_id** | **function** |
| --- | --- | --- | --- | --- | --- | --- | --- | --- | --- |
| CP018454.1 | 575140 | G | A | 0 | 0 | 1 | 0 | non-coded |  |
| CP018454.1 | 1519002 | T | C | 0 | 0 | 1 | 1 | non-coded |  |
| CP018454.1 | 2875490 | G | A | 0 | 1 | 0 | 0 | gene2894 | L-serine dehydratase, beta subunit (EC 4.3.1.17) / L-serine dehydratase, alpha subunit (EC 4.3.1.17) |
| CP018454.1 | 2998674 | A | G | 0 | 0 | 0 | 1 | non-coded |  |
| CP018454.1 | 3727894 | T | C | 0 | 1 | 0 | 0 | gene3778 | Transposase |
| CP018454.1 | 3727915 | C | T | 0 | 1 | 0 | 0 |  |  |
| CP018454.1 | 3727918 | G | T | 0 | 1 | 0 | 0 |  |  |
| CP018454.1 | 3727944 | A | G | 0 | 1 | 0 | 0 |  |  |
| CP018454.1 | 3727954 | G | A | 0 | 1 | 0 | 0 |  |  |
| CP018454.1 | 3728680 | G | C | 0 | 1 | 0 | 0 |  |  |
| CP018454.1 | 4190742 | G | T | 1 | 0 | 0 | 0 | gene4245 | DNA-directed RNA polymerase beta subunit (EC 2.7.7.6) |
| CP018454.1 | 4253589 | G | A | 0 | 0 | 0 | 1 | gene4303 | Putative inner membrane protein |
| CP018454.1 | 4516959 | C | T | 0 | 0 | 0 | 1 | gene4574 | hypothetical protein |
